# Supplementary material for: Risk Assessment of Toxic Heavy Metal Exposure in Selected Seafood Species from Thailand
Source: Foods. 2025 Oct 30;14(21):3725. doi: 10.3390/foods14213725 (PMC12607330; doi:10.3390/foods14213725)
Supplement: Supplementary file 1 [file foods-14-03725-s001.zip › foods-3940486-supplementary.pdf]

Table S1. Margin of exposure of arsenic from each seafood species (Per eater-only).

Type of sample		Cooking method	Food consumption	Age group (Year old)					
				3 to 5.9	6 to 12.9	13 to 17.9	18 to 34.9	35 to 64.9	65 and older
Shrimp and prawn (captured)	Pacific white shrimp	Boiled	average	**0.2**	**0.2**	**0.4**	**0.5**	**0.5**	**0.6**
			97.5th percentile	**0.08**	**0.1**	**0.2**	**0.3**	**0.3**	**0.2**
		Fried	average	**0.2**	**0.3**	**0.5**	**0.5**	**0.6**	**0.7**
			97.5th percentile	**0.09**	**0.2**	**0.3**	**0.3**	**0.3**	**0.3**
		Grilled	average	**0.2**	**0.3**	**0.5**	**0.5**	**0.6**	**0.7**
			97.5th percentile	**0.09**	**0.2**	**0.3**	**0.3**	**0.3**	**0.3**
	Banana prawn	Boiled	average	**0.2**	**0.2**	**0.4**	**0.4**	**0.5**	**0.5**
			97.5th percentile	**0.1**	**0.1**	**0.2**	**0.3**	**0.3**	**0.2**
		Fried	average	**0.2**	**0.3**	**0.4**	**0.5**	**0.6**	**0.6**
			97.5th percentile	**0.1**	**0.2**	**0.3**	**0.3**	**0.3**	**0.3**
		Grilled	average	**0.2**	**0.3**	**0.4**	**0.5**	**0.6**	**0.6**
			97.5th percentile	**0.1**	**0.2**	**0.3**	**0.3**	**0.3**	**0.3**
	Giant Tiger Prawn	Boiled	average	**0.2**	**0.3**	**0.5**	**0.6**	**0.7**	**0.8**
			97.5th percentile	**0.1**	**0.2**	**0.3**	**0.4**	**0.4**	**0.3**
		Fried	average	**0.2**	**0.3**	**0.5**	**0.5**	**0.6**	**0.7**
			97.5th percentile	**0.1**	**0.2**	**0.3**	**0.3**	**0.3**	**0.3**
		Grilled	average	**0.2**	**0.3**	**0.5**	**0.5**	**0.6**	**0.7**
			97.5th percentile	**0.1**	**0.2**	**0.3**	**0.3**	**0.3**	**0.3**
	Ornate rock lobster	Boiled	average	**0.03**	**0.04**	**0.08**	**0.08**	**0.10**	**0.1**
			97.5th percentile	**0.01**	**0.03**	**0.04**	**0.05**	**0.05**	**0.05**
		Fried	average	**0.04**	**0.06**	**0.10**	**0.11**	**0.14**	**0.1**
			97.5th percentile	**0.02**	**0.04**	**0.06**	**0.07**	**0.07**	**0.06**
		Grilled	average	**0.03**	**0.04**	**0.06**	**0.07**	**0.09**	**0.1**
			97.5th percentile	**0.01**	**0.02**	**0.04**	**0.04**	**0.04**	**0.04**
Crabs (captured)	Musk Crab	Boiled	average	**0.03**	**0.04**	**0.06**	**0.06**	**0.05**	**0.07**
			97.5th percentile	**0.02**	**0.02**	**0.02**	**0.02**	**0.02**	**0.03**
		Fried	average	**0.02**	**0.03**	**0.04**	**0.04**	**0.04**	**0.04**
			97.5th percentile	**0.01**	**0.01**	**0.01**	**0.01**	**0.01**	**0.02**
		Grilled	average	**0.02**	**0.03**	**0.05**	**0.05**	**0.04**	**0.06**
			97.5th percentile	**0.01**	**0.01**	**0.01**	**0.01**	**0.02**	**0.02**
	Blue crab	Boiled	average	**0.2**	**0.3**	**0.4**	**0.4**	**0.4**	**0.5**
			97.5th percentile	**0.12**	**0.11**	**0.12**	**0.11**	**0.12**	**0.2**
		Fried	average	**0.1**	**0.2**	**0.3**	**0.3**	**0.3**	**0.3**
			97.5th percentile	**0.08**	**0.08**	**0.09**	**0.08**	**0.09**	**0.1**
		Grilled	average	**0.1**	**0.2**	**0.3**	**0.3**	**0.3**	**0.3**
			97.5th percentile	**0.08**	**0.08**	**0.09**	**0.08**	**0.09**	**0.1**

Table S1. Margin of exposure of arsenic from each seafood species (Per eater-only). Cont.

Type of sample		Cooking method	Food consumption	Age group (Year old)					
				3 to 5.9	6 to 12.9	13 to 17.9	18 to 34.9	35 to 64.9	65 and older
Crabs (captured)	Serrated Mud Crab	Boiled	average	**0.2**	**0.3**	**0.4**	**0.4**	**0.4**	**0.5**
			97.5th percentile	**0.13**	**0.13**	**0.14**	**0.12**	**0.14**	**0.2**
		Fried	average	**0.2**	**0.3**	**0.4**	**0.4**	**0.4**	**0.5**
			97.5th percentile	**0.13**	**0.13**	**0.14**	**0.12**	**0.14**	**0.2**
		Grilled	average	**0.2**	**0.3**	**0.4**	**0.4**	**0.4**	**0.5**
			97.5th percentile	**0.13**	**0.13**	**0.14**	**0.12**	**0.14**	**0.2**
	Red frog crab	Boiled	average	**0.3**	**0.4**	**0.6**	**0.6**	**0.6**	**0.7**
			97.5th percentile	**0.2**	**0.2**	**0.2**	**0.2**	**0.2**	**0.3**
		Fried	average	**0.2**	**0.4**	**0.5**	**0.5**	**0.5**	**0.6**
			97.5th percentile	**0.2**	**0.2**	**0.2**	**0.2**	**0.2**	**0.3**
		Grilled	average	**0.3**	**0.4**	**0.6**	**0.6**	**0.6**	**0.7**
			97.5th percentile	**0.2**	**0.2**	**0.2**	**0.2**	**0.2**	**0.3**
Squids (captured)	Splendid squid	Boiled	average	**0.14**	**0.2**	**0.3**	**0.3**	**0.4**	**0.5**
			97.5th percentile	**0.05**	**0.09**	**0.14**	**0.2**	**0.2**	**0.2**
		Fried	average	**0.10**	**0.1**	**0.2**	**0.2**	**0.3**	**0.3**
			97.5th percentile	**0.04**	**0.06**	**0.10**	**0.1**	**0.1**	**0.1**
		Grilled	average	**0.15**	**0.2**	**0.3**	**0.4**	**0.5**	**0.5**
			97.5th percentile	**0.06**	**0.10**	**0.16**	**0.2**	**0.2**	**0.2**
	Cuttlefish	Boiled	average	**0.1**	**0.2**	**0.3**	**0.4**	**0.4**	**0.5**
			97.5th percentile	**0.06**	**0.10**	**0.2**	**0.2**	**0.2**	**0.2**
		Fried	average	**0.1**	**0.2**	**0.2**	**0.3**	**0.3**	**0.4**
			97.5th percentile	**0.04**	**0.07**	**0.1**	**0.1**	**0.1**	**0.1**
		Grilled	average	**0.1**	**0.2**	**0.3**	**0.4**	**0.4**	**0.5**
			97.5th percentile	**0.06**	**0.10**	**0.2**	**0.2**	**0.2**	**0.2**
	Bigfin reef squid	Boiled	average	**0.07**	**0.09**	**0.1**	**0.2**	**0.2**	**0.2**
			97.5th percentile	**0.03**	**0.04**	**0.07**	**0.08**	**0.08**	**0.08**
		Fried	average	**0.06**	**0.08**	**0.1**	**0.1**	**0.2**	**0.2**
			97.5th percentile	**0.02**	**0.04**	**0.06**	**0.07**	**0.07**	**0.07**
		Grilled	average	**0.06**	**0.08**	**0.1**	**0.1**	**0.2**	**0.2**
			97.5th percentile	**0.02**	**0.04**	**0.06**	**0.07**	**0.07**	**0.07**
Shellfish (captured)	Razor clam	Boiled	average	**0.29**	**0.58**	**0.88**	**0.73**	**1.01**	**1.25**
			97.5th percentile	**0.14**	**0.28**	**0.44**	**0.35**	**0.53**	**0.92**
		Fried	average	**0.25**	**0.51**	**0.77**	**0.64**	**0.88**	1.09
			97.5th percentile	**0.13**	**0.24**	**0.39**	**0.31**	**0.46**	**0.81**
		Grilled	average	**0.25**	**0.51**	**0.77**	**0.64**	**0.88**	1.09
			97.5th percentile	**0.13**	**0.24**	**0.39**	**0.31**	**0.46**	**0.81**
	Oysters	Boiled	average	**0.22**	**0.26**	**0.36**	**0.42**	**0.50**	**0.56**
			97.5th percentile	**0.06**	**0.22**	**0.18**	**0.21**	**0.21**	**0.37**
		Fried	average	**0.18**	**0.21**	**0.29**	**0.33**	**0.40**	**0.45**
			97.5th percentile	**0.05**	**0.18**	**0.14**	**0.17**	**0.17**	**0.30**
		Grilled	average	**0.13**	**0.15**	**0.22**	**0.25**	**0.30**	**0.34**
			97.5th percentile	**0.03**	**0.13**	**0.11**	**0.13**	**0.13**	**0.22**

Table S1. Margin of exposure of arsenic from each seafood species (Per eater-only). Cont.

Type of sample		Cooking method	Food consumption	Age group (Year old)					
				3 to 5.9	6 to 12.9	13 to 17.9	18 to 34.9	35 to 64.9	65 and older
Shellfish (captured)	Cockle	Boiled	average	0.20	0.30	0.45	0.50	0.53	0.62
			97.5th percentile	0.09	0.09	0.29	0.17	0.23	0.30
		Fried	average	0.16	0.24	0.36	0.40	0.42	0.49
			97.5th percentile	0.08	0.07	0.23	0.14	0.18	0.24
		Grilled	average	0.08	0.12	0.18	0.20	0.21	0.25
			97.5th percentile	0.04	0.04	0.12	0.07	0.09	0.12
	Clam	Boiled	average	1.65	2.50	3.54	3.89	4.59	5.33
			97.5th percentile	0.66	1.27	1.36	2.40	2.42	2.83
		Fried	average	1.10	1.67	2.36	2.59	3.06	3.56
			97.5th percentile	0.44	0.85	0.90	1.60	1.61	1.89
		Grilled	average	1.37	2.09	2.95	3.24	3.83	4.44
			97.5th percentile	0.55	1.06	1.13	2.00	2.01	2.36
	Mussels	Boiled	average	0.31	0.43	0.65	0.69	0.73	0.83
			97.5th percentile	0.13	0.25	0.40	0.24	0.19	0.42
		Fried	average	0.18	0.26	0.39	0.41	0.44	0.50
			97.5th percentile	0.08	0.15	0.24	0.14	0.11	0.25
		Grilled	average	0.18	0.26	0.39	0.41	0.44	0.50
			97.5th percentile	0.08	0.15	0.24	0.14	0.11	0.25
	Wedge shell	Boiled	average	0.02	0.04	0.06	0.05	0.07	0.09
			97.5th percentile	0.01	0.02	0.03	0.03	0.04	0.07
		Fried	average	0.02	0.03	0.05	0.04	0.06	0.08
			97.5th percentile	0.01	0.02	0.03	0.02	0.03	0.06
		Grilled	average	0.02	0.04	0.06	0.05	0.07	0.09
			97.5th percentile	0.01	0.02	0.03	0.03	0.04	0.07
	Indo-Pacific horseshoe crab (eggs)	Boiled	average	0.16	0.23	0.37	0.42	0.50	0.45
			97.5th percentile	0.08	0.08	0.12	0.15	0.29	0.13
		Fried	average	0.13	0.18	0.29	0.33	0.39	0.35
			97.5th percentile	0.06	0.06	0.10	0.11	0.23	0.10
		Grilled	average	0.15	0.21	0.33	0.38	0.44	0.40
			97.5th percentile	0.07	0.07	0.11	0.13	0.26	0.11
Marine fish (farmed)	Northern whiting fish	Boiled	average	0.41	0.56	0.89	0.95	1.09	1.17
			97.5th percentile	0.24	0.24	0.38	0.45	0.45	0.41
		Fried	average	0.23	0.31	0.49	0.53	0.61	0.65
			97.5th percentile	0.14	0.13	0.21	0.25	0.25	0.23
		Grilled	average	0.28	0.37	0.59	0.64	0.73	0.78
			97.5th percentile	0.16	0.16	0.25	0.30	0.30	0.27
	Silver pomfret	Boiled	average	2.1	2.9	4.5	4.9	5.6	6.0
			97.5th percentile	1.2	1.2	1.9	2.3	2.3	2.1
		Fried	average	1.2	1.6	2.5	2.7	3.1	3.3
			97.5th percentile	0.7	0.7	1.1	1.3	1.3	1.2
		Grilled	average	1.6	2.2	3.5	3.8	4.3	4.6
			97.5th percentile	1.0	0.9	1.5	1.8	1.8	1.6

Table S2. Hazard quotient of cadmium from each seafood species (Per capita).

Type of sample		Cooking method	Food consumption	Age group (Year old)					
				3 to 5.9	6 to 12.9	13 to 17.9	18 to 34.9	35 to 64.9	65 and older
Shrimp and prawn (captured)	Pacific white shrimp	Boiled	average	0.002	0.001	0.001	0.001	0.0004	0.0002
			97.5th percentile	0.014	0.011	0.004	0.004	0.003	0.002
		Fried	average	0.002	0.001	0.001	0.001	0.0003	0.0002
			97.5th percentile	0.012	0.009	0.004	0.003	0.002	0.002
		Grilled	average	0.002	0.001	0.001	0.001	0.0003	0.0002
			97.5th percentile	0.012	0.009	0.004	0.003	0.002	0.002
	Banana prawn	Boiled	average	0.017	0.012	0.007	0.005	0.003	0.002
			97.5th percentile	0.121	0.094	0.039	0.033	0.025	0.019
		Fried	average	0.015	0.010	0.006	0.005	0.003	0.002
			97.5th percentile	0.104	0.081	0.034	0.028	0.021	0.016
		Grilled	average	0.015	0.010	0.006	0.005	0.003	0.002
			97.5th percentile	0.104	0.081	0.034	0.028	0.021	0.016
	Giant Tiger Prawn	Boiled	average	0.018	0.012	0.008	0.006	0.003	0.002
			97.5th percentile	0.127	0.099	0.041	0.035	0.026	0.020
		Fried	average	0.021	0.015	0.009	0.007	0.004	0.003
			97.5th percentile	0.148	0.115	0.048	0.041	0.030	0.023
		Grilled	average	0.021	0.015	0.009	0.007	0.004	0.003
			97.5th percentile	0.148	0.115	0.048	0.041	0.030	0.023
	Ornate rock lobster	Boiled	average	0.021	0.015	0.009	0.007	0.004	0.003
			97.5th percentile	0.148	0.115	0.048	0.041	0.030	0.023
		Fried	average	0.125	0.085	0.053	0.039	0.023	0.015
			97.5th percentile	0.868	0.673	0.280	0.237	0.177	0.134
		Grilled	average	0.200	0.136	0.084	0.063	0.036	0.023
			97.5th percentile	**1.388**	**1.076**	0.448	0.379	0.283	0.215
Crabs (captured)	Musk Crab	Boiled	average	0.095	0.058	0.036	0.044	0.032	0.011
			97.5th percentile	0.856	0.619	0.276	0.351	0.232	0.011
		Fried	average	0.143	0.086	0.053	0.066	0.048	0.017
			97.5th percentile	**1.283**	0.928	0.414	0.526	0.348	0.017
		Grilled	average	0.114	0.069	0.043	0.053	0.038	0.014
			97.5th percentile	**1.027**	0.742	0.332	0.421	0.279	0.014
	Blue crab	Boiled	average	0.029	0.017	0.011	0.013	0.010	0.003
			97.5th percentile	0.259	0.188	0.084	0.106	0.070	0.037
		Fried	average	0.040	0.024	0.015	0.019	0.013	0.005
			97.5th percentile	0.363	0.263	0.117	0.149	0.099	0.052
		Grilled	average	0.040	0.024	0.015	0.019	0.013	0.005
			97.5th percentile	0.363	0.263	0.117	0.149	0.099	0.052

Table S2. Hazard quotient of cadmium from each seafood species (Per capita). Cont.

Type of sample		Cooking method	Food consumption	Age group (Year old)					
				3 to 5.9	6 to 12.9	13 to 17.9	18 to 34.9	35 to 64.9	65 and older
Crabs (captured)	Serrated Mud Crab	Boiled	average	0.025	0.015	0.009	0.012	0.008	0.003
			97.5th percentile	0.224	0.162	0.072	0.092	0.061	0.032
		Fried	average	0.025	0.015	0.009	0.012	0.008	0.003
			97.5th percentile	0.224	0.162	0.072	0.092	0.061	0.032
		Grilled	average	0.025	0.015	0.009	0.012	0.008	0.003
			97.5th percentile	0.224	0.162	0.072	0.092	0.061	0.032
	Red frog crab	Boiled	average	0.059	0.036	0.022	0.027	0.020	0.007
			97.5th percentile	0.528	0.382	0.171	0.217	0.143	0.076
		Fried	average	0.069	0.042	0.026	0.032	0.023	0.008
			97.5th percentile	0.617	0.446	0.199	0.253	0.167	0.089
		Grilled	average	0.059	0.036	0.022	0.027	0.020	0.007
			97.5th percentile	0.528	0.382	0.171	0.217	0.143	0.076
Squids (captured)	Splendid squid	Boiled	average	0.019	0.016	0.012	0.010	0.004	0.002
			97.5th percentile	0.138	0.095	0.089	0.063	0.025	0.014
		Fried	average	0.026	0.022	0.016	0.014	0.006	0.003
			97.5th percentile	0.193	0.133	0.125	0.088	0.035	0.020
		Grilled	average	0.016	0.014	0.010	0.009	0.004	0.002
			97.5th percentile	0.121	0.083	0.078	0.055	0.022	0.012
	Cuttlefish	Boiled	average	0.109	0.092	0.067	0.057	0.025	0.011
			97.5th percentile	0.798	0.550	0.516	0.364	0.145	0.082
		Fried	average	0.152	0.129	0.093	0.080	0.035	0.015
			97.5th percentile	**1.118**	0.770	0.722	0.509	0.202	0.115
		Grilled	average	0.109	0.092	0.067	0.057	0.025	0.011
			97.5th percentile	0.798	0.550	0.516	0.364	0.145	0.082
	Bigfin reef squid	Boiled	average	0.004	0.004	0.003	0.002	0.001	0.000
			97.5th percentile	0.031	0.021	0.020	0.014	0.006	0.003
		Fried	average	0.005	0.004	0.003	0.003	0.001	0.0005
			97.5th percentile	0.036	0.025	0.023	0.016	0.006	0.004
		Grilled	average	0.005	0.004	0.003	0.003	0.001	0.0005
			97.5th percentile	0.036	0.025	0.023	0.016	0.006	0.004
Shellfish (captured)	Razor clam	Boiled	average	0.001	0.002	0.0005	0.0005	0.0004	0.0003
			97.5th percentile	-	0.006	-	-	-	-
		Fried	average	0.001	0.002	0.001	0.001	0.0005	0.0004
			97.5th percentile	-	0.007	-	-	-	-
		Grilled	average	0.001	0.002	0.001	0.001	0.0005	0.0004
			97.5th percentile	-	0.007	-	-	-	-
	Oysters	Boiled	average	0.005	0.010	0.041	0.042	0.013	0.008
			97.5th percentile	-	-	0.343	0.435	0.144	-
		Fried	average	0.006	0.012	0.051	0.052	0.016	0.010
			97.5th percentile	-	-	0.429	0.544	0.180	-
		Grilled	average	0.008	0.017	0.068	0.069	0.022	0.014
			97.5th percentile	-	-	0.571	0.725	0.240	-

Table S2. Hazard quotient of cadmium from each seafood species (Per capita). Cont.

Type of sample		Cooking method	Food consumption	Age group (Year old)					
				3 to 5.9	6 to 12.9	13 to 17.9	18 to 34.9	35 to 64.9	65 and older
Shellfish (captured)	Cockle	Boiled	average	0.184	0.167	0.111	0.107	0.052	0.019
			97.5th percentile	0.974	**2.014**	**1.258**	**1.065**	0.529	0.151
		Fried	average	0.230	0.209	0.139	0.133	0.066	0.023
			97.5th percentile	**1.217**	**2.517**	**1.573**	**1.331**	0.662	0.188
		Grilled	average	0.459	0.418	0.278	0.267	0.131	0.047
			97.5th percentile	**2.434**	**5.034**	**3.146**	**2.662**	**1.323**	0.376
	Clam	Boiled	average	0.00002	0.00002	0.00001	0.00002	0.00001	0.00001
			97.5th percentile	0.0003	0.0003	0.0002	0.0002	0.0001	0.0001
		Fried	average	0.00003	0.00003	0.00002	0.00003	0.00002	0.00001
			97.5th percentile	0.0004	0.0004	0.0003	0.0003	0.0002	0.0001
		Grilled	average	0.00003	0.00003	0.00002	0.00002	0.00002	0.00001
			97.5th percentile	0.0003	0.0003	0.0002	0.0002	0.0002	0.0001
	Mussels	Boiled	average	0.026	0.021	0.012	0.018	0.010	0.004
			97.5th percentile	0.359	0.186	0.116	0.147	0.097	0.039
		Fried	average	0.044	0.036	0.020	0.030	0.016	0.006
			97.5th percentile	0.598	0.309	0.193	0.245	0.162	0.065
		Grilled	average	0.044	0.036	0.020	0.030	0.016	0.006
			97.5th percentile	0.598	0.309	0.193	0.245	0.162	0.065
	Wedge shell	Boiled	average	0.034	0.071	0.017	0.016	0.014	0.011
			97.5th percentile	-	0.208	-	-	-	-
		Fried	average	0.041	0.085	0.020	0.020	0.017	0.013
			97.5th percentile	-	0.250	-	-	-	-
		Grilled	average	0.034	0.071	0.017	0.016	0.014	0.011
			97.5th percentile	-	0.208	-	-	-	-
	Indo-Pacific horseshoe crab (eggs)	Boiled	average	0.004	0.010	0.004	0.004	0.003	0.003
			97.5th percentile	0.054	0.121	0.035	0.038	0.030	0.034
		Fried	average	0.006	0.012	0.004	0.005	0.003	0.003
			97.5th percentile	0.070	0.156	0.045	0.049	0.039	0.044
		Grilled	average	0.005	0.010	0.004	0.004	0.003	0.003
			97.5th percentile	0.061	0.136	0.040	0.043	0.034	0.038
Marine fish (farmed)	Northern whiting fish	Boiled	average	0.0002	0.0001	0.0001	0.0001	0.0001	0.0001
			97.5th percentile	0.002	0.001	0.001	0.002	0.001	0.003
		Fried	average	0.0003	0.0002	0.0001	0.0002	0.0002	0.0001
			97.5th percentile	0.003	0.002	0.002	0.003	0.002	0.005
		Grilled	average	0.0003	0.0002	0.0001	0.0002	0.0002	0.0001
			97.5th percentile	0.003	0.001	0.001	0.003	0.001	0.004
	Silver pomfret	Boiled	average	0.001	0.0005	0.0003	0.001	0.0005	0.0003
			97.5th percentile	0.008	0.004	0.004	0.008	0.004	0.003
		Fried	average	0.001	0.001	0.0005	0.001	0.001	0.001
			97.5th percentile	0.015	0.008	0.007	0.014	0.008	0.005
		Grilled	average	0.001	0.001	0.0003	0.001	0.001	0.0004
			97.5th percentile	0.011	0.005	0.005	0.010	0.006	0.003

Table S3. Hazard quotient of cadmium from each seafood species (Per eater-only).

Type of sample		Cooking method	Food consumption	Age group (Year old)					
				3 to 5.9	6 to 12.9	13 to 17.9	18 to 34.9	35 to 64.9	65 and older
Shrimp and prawn (captured)	Pacific white shrimp	Boiled	average	0.022	0.015	0.009	0.008	0.007	0.006
			97.5th percentile	0.048	0.025	0.015	0.013	0.013	0.015
		Fried	average	0.019	0.013	0.008	0.007	0.006	0.005
			97.5th percentile	0.041	0.021	0.013	0.011	0.011	0.013
		Grilled	average	0.019	0.013	0.008	0.007	0.006	0.005
			97.5th percentile	0.041	0.021	0.013	0.011	0.011	0.013
	Banana prawn	Boiled	average	0.197	0.137	0.079	0.071	0.059	0.055
			97.5th percentile	0.424	0.219	0.137	0.116	0.115	0.131
		Fried	average	0.169	0.117	0.067	0.061	0.050	0.047
			97.5th percentile	0.363	0.188	0.117	0.099	0.099	0.112
		Grilled	average	0.169	0.117	0.067	0.061	0.050	0.047
			97.5th percentile	0.363	0.188	0.117	0.099	0.099	0.112
	Giant Tiger Prawn	Boiled	average	0.207	0.144	0.083	0.075	0.062	0.057
			97.5th percentile	0.445	0.230	0.144	0.122	0.121	0.138
		Fried	average	0.241	0.168	0.096	0.087	0.072	0.067
			97.5th percentile	0.519	0.268	0.168	0.142	0.141	0.161
		Grilled	average	0.241	0.168	0.096	0.087	0.072	0.067
			97.5th percentile	0.519	0.268	0.168	0.142	0.141	0.161
	Ornate rock lobster	Boiled	average	0.241	0.168	0.096	0.087	0.072	0.067
			97.5th percentile	0.519	0.268	0.168	0.142	0.141	0.161
		Fried	average	**1.412**	0.981	0.563	0.511	0.421	0.391
			97.5th percentile	**3.037**	**1.569**	0.981	0.830	0.825	0.939
		Grilled	average	**2.259**	**1.569**	0.902	0.818	0.674	0.626
			97.5th percentile	**4.859**	**2.511**	**1.569**	**1.328**	**1.319**	**1.503**
Crabs (captured)	Musk Crab	Boiled	average	**4.099**	**2.620**	**1.841**	**1.881**	**1.945**	**1.538**
			97.5th percentile	**5.985**	**6.186**	**5.798**	**6.543**	**5.688**	**1.538**
		Fried	average	**6.148**	**3.930**	**2.761**	**2.821**	**2.918**	**2.307**
			97.5th percentile	**8.978**	**9.279**	**8.697**	**9.814**	**8.532**	**2.307**
		Grilled	average	**4.918**	**3.144**	**2.209**	**2.257**	**2.334**	**1.845**
			97.5th percentile	**7.182**	**7.423**	**6.958**	**7.852**	**6.826**	**1.845**
	Blue crab	Boiled	average	**1.243**	0.794	0.558	0.570	0.590	0.466
			97.5th percentile	**1.815**	**1.875**	**1.758**	**1.984**	**1.724**	**1.122**
		Fried	average	**1.740**	**1.112**	0.781	0.798	0.826	0.653
			97.5th percentile	**2.540**	**2.626**	**2.461**	**2.777**	**2.414**	**1.571**
		Grilled	average	**1.740**	**1.112**	0.781	0.798	0.826	0.653
			97.5th percentile	**2.540**	**2.626**	**2.461**	**2.777**	**2.414**	**1.571**

Table S3. Hazard quotient of cadmium from each seafood species (Per eater-only). Cont.

Type of sample		Cooking method	Food consumption	Age group (Year old)					
				3 to 5.9	6 to 12.9	13 to 17.9	18 to 34.9	35 to 64.9	65 and older
Crabs (captured)	Serrated Mud Crab	Boiled	average	**1.071**	0.684	0.481	0.491	0.508	0.402
			97.5th percentile	**1.563**	**1.616**	**1.515**	**1.709**	**1.486**	0.967
		Fried	average	**1.071**	0.684	0.481	0.491	0.508	0.402
			97.5th percentile	**1.563**	**1.616**	**1.515**	**1.709**	**1.486**	0.967
		Grilled	average	**1.071**	0.684	0.481	0.491	0.508	0.402
			97.5th percentile	**1.563**	**1.616**	**1.515**	**1.709**	**1.486**	0.967
	Red frog crab	Boiled	average	**2.531**	**1.618**	**1.137**	**1.162**	**1.201**	0.950
			97.5th percentile	**3.697**	**3.821**	**3.581**	**4.041**	**3.513**	**2.287**
		Fried	average	**2.953**	**1.888**	**1.326**	**1.355**	**1.402**	**1.108**
			97.5th percentile	**4.313**	**4.458**	**4.178**	**4.715**	**4.099**	**2.668**
		Grilled	average	**2.531**	**1.618**	**1.137**	**1.162**	**1.201**	0.950
			97.5th percentile	**3.697**	**3.821**	**3.581**	**4.041**	**3.513**	**2.287**
Squids (captured)	Splendid squid	Boiled	average	0.211	0.150	0.093	0.087	0.073	0.061
			97.5th percentile	0.537	0.333	0.208	0.176	0.175	0.166
		Fried	average	0.296	0.210	0.131	0.122	0.102	0.085
			97.5th percentile	0.752	0.467	0.292	0.247	0.245	0.233
		Grilled	average	0.185	0.131	0.082	0.076	0.064	0.053
			97.5th percentile	0.470	0.292	0.182	0.154	0.153	0.145
	Cuttlefish	Boiled	average	**1.221**	0.865	0.539	0.503	0.419	0.351
			97.5th percentile	**3.105**	**1.925**	**1.203**	**1.018**	**1.012**	0.960
		Fried	average	**1.710**	**1.211**	0.755	0.704	0.587	0.492
			97.5th percentile	**4.346**	**2.695**	**1.684**	**1.425**	**1.416**	**1.344**
		Grilled	average	**1.221**	0.865	0.539	0.503	0.419	0.351
			97.5th percentile	**3.105**	**1.925**	**1.203**	**1.018**	**1.012**	0.960
	Bigfin reef squid	Boiled	average	0.047	0.033	0.021	0.019	0.016	0.013
			97.5th percentile	0.119	0.074	0.046	0.039	0.039	0.037
		Fried	average	0.055	0.039	0.024	0.023	0.019	0.016
			97.5th percentile	0.139	0.086	0.054	0.046	0.045	0.043
		Grilled	average	0.055	0.039	0.024	0.023	0.019	0.016
			97.5th percentile	0.139	0.086	0.054	0.046	0.045	0.043
Shellfish (captured)	Razor clam	Boiled	average	0.696	0.347	0.229	0.275	0.199	0.161
			97.5th percentile	**1.405**	0.726	0.454	0.576	0.382	0.217
		Fried	average	0.796	0.397	0.261	0.314	0.227	0.184
			97.5th percentile	**1.606**	0.830	0.519	0.658	0.436	0.248
		Grilled	average	0.796	0.397	0.261	0.314	0.227	0.184
			97.5th percentile	**1.606**	0.830	0.519	0.658	0.436	0.248
	Oysters	Boiled	average	**8.236**	**7.125**	**5.101**	**4.391**	**3.686**	**3.281**
			97.5th percentile	**31.855**	**8.231**	**10.286**	**8.706**	**8.649**	**4.926**
		Fried	average	**10.296**	**8.906**	**6.376**	**5.489**	**4.608**	**4.101**
			97.5th percentile	**39.819**	**10.289**	**12.858**	**10.882**	**10.812**	**6.158**
		Grilled	average	**13.727**	**11.875**	**8.502**	**7.318**	**6.144**	**5.468**
			97.5th percentile	**53.091**	**13.718**	**17.144**	**14.509**	**14.416**	**8.211**

Table S3. Hazard quotient of cadmium from each seafood species (Per eater-only). Cont.

Type of sample		Cooking method	Food consumption	Age group (Year old)					
				3 to 5.9	6 to 12.9	13 to 17.9	18 to 34.9	35 to 64.9	65 and older
Shellfish (captured)	Cockle	Boiled	average	**6.357**	**4.323**	**2.823**	**2.576**	**2.427**	**2.075**
			97.5th percentile	**13.639**	**14.097**	**4.404**	**7.455**	**5.555**	**4.219**
		Fried	average	**7.947**	**5.404**	**3.529**	**3.220**	**3.034**	**2.594**
			97.5th percentile	**17.049**	**17.621**	**5.505**	**9.319**	**6.944**	**5.273**
		Grilled	average	**15.893**	**10.807**	**7.057**	**6.440**	**6.068**	**5.188**
			97.5th percentile	**34.098**	**35.242**	**11.011**	**18.637**	**13.888**	**10.547**
	Clam	Boiled	average	0.003	0.002	0.001	0.001	0.001	0.001
			97.5th percentile	0.007	0.004	0.003	0.002	0.002	0.002
		Fried	average	0.004	0.003	0.002	0.002	0.002	0.001
			97.5th percentile	0.011	0.006	0.005	0.003	0.003	0.003
		Grilled	average	0.003	0.002	0.002	0.001	0.001	0.001
			97.5th percentile	0.009	0.004	0.004	0.002	0.002	0.002
	Mussels	Boiled	average	**1.058**	0.743	0.500	0.470	0.442	0.388
			97.5th percentile	**2.513**	**1.299**	0.812	**1.374**	**1.706**	0.777
		Fried	average	**1.764**	**1.238**	0.833	0.784	0.737	0.646
			97.5th percentile	**4.188**	**2.165**	**1.353**	**2.289**	**2.843**	**1.296**
		Grilled	average	**1.764**	**1.238**	0.833	0.784	0.737	0.646
			97.5th percentile	**4.188**	**2.165**	**1.353**	**2.289**	**2.843**	**1.296**
	Wedge shell	Boiled	average	**23.766**	**11.843**	**7.801**	**9.385**	**6.785**	**5.486**
			97.5th percentile	**47.959**	**24.784**	**15.487**	**19.660**	**13.022**	**7.417**
		Fried	average	**28.519**	**14.212**	**9.362**	**11.262**	**8.143**	**6.583**
			97.5th percentile	**57.551**	**29.741**	**18.584**	**23.592**	**15.627**	**8.900**
		Grilled	average	**23.766**	**11.843**	**7.801**	**9.385**	**6.785**	**5.486**
			97.5th percentile	**47.959**	**24.784**	**15.487**	**19.660**	**13.022**	**7.417**
	Indo-Pacific horseshoe crab (eggs)	Boiled	average	0.795	0.554	0.351	0.362	0.262	0.290
			97.5th percentile	**1.640**	**1.695**	**1.059**	**1.059**	0.448	**1.014**
		Fried	average	**1.023**	0.712	0.451	0.466	0.337	0.372
			97.5th percentile	**2.108**	**2.179**	**1.362**	**1.362**	0.576	**1.304**
		Grilled	average	0.895	0.623	0.395	0.408	0.294	0.326
			97.5th percentile	**1.845**	**1.907**	**1.191**	**1.191**	0.504	**1.141**
Marine fish (farmed)	Northern whiting fish	Boiled	average	0.008	0.006	0.004	0.003	0.003	0.003
			97.5th percentile	0.013	0.013	0.008	0.007	0.007	0.034
		Fried	average	0.014	0.010	0.006	0.006	0.005	0.005
			97.5th percentile	0.023	0.024	0.015	0.013	0.012	0.062
		Grilled	average	0.011	0.008	0.005	0.005	0.004	0.004
			97.5th percentile	0.019	0.020	0.012	0.010	0.010	0.052
	Silver pomfret	Boiled	average	0.034	0.025	0.016	0.015	0.013	0.012
			97.5th percentile	0.058	0.060	0.037	0.032	0.031	0.034
		Fried	average	0.062	0.045	0.029	0.027	0.023	0.022
			97.5th percentile	0.104	0.108	0.067	0.057	0.057	0.062
		Grilled	average	0.044	0.032	0.021	0.019	0.017	0.016
			97.5th percentile	0.074	0.077	0.048	0.041	0.040	0.044

Table S4. Hazard quotient of mercury from each seafood species (Per capita).

Type of sample		Cooking method	Food consumption	Age group (Year old)					
				3 to 5.9	6 to 12.9	13 to 17.9	18 to 34.9	35 to 64.9	65 and older
Shrimp and prawn (captured)	Pacific white shrimp	Boiled	average	0.001	0.0005	0.0003	0.0002	0.0001	0.0001
			97.5th percentile	0.005	0.004	0.001	0.001	0.001	0.001
		Fried	average	0.001	0.0005	0.0003	0.0002	0.0001	0.0001
			97.5th percentile	0.005	0.004	0.001	0.001	0.001	0.001
		Grilled	average	0.001	0.0004	0.0002	0.0002	0.0001	0.0001
			97.5th percentile	0.004	0.003	0.001	0.001	0.001	0.001
	Banana prawn	Boiled	average	0.001	0.0005	0.0003	0.0002	0.0001	0.0001
			97.5th percentile	0.005	0.004	0.001	0.001	0.001	0.001
		Fried	average	0.001	0.0005	0.0003	0.0002	0.0001	0.0001
			97.5th percentile	0.005	0.004	0.001	0.001	0.001	0.001
		Grilled	average	0.001	0.0004	0.0002	0.0002	0.0001	0.0001
			97.5th percentile	0.004	0.003	0.001	0.001	0.001	0.001
	Giant Tiger Prawn	Boiled	average	0.001	0.0004	0.0002	0.0002	0.0001	0.0001
			97.5th percentile	0.004	0.003	0.001	0.001	0.001	0.001
		Fried	average	0.001	0.0004	0.0002	0.0002	0.0001	0.0001
			97.5th percentile	0.004	0.003	0.001	0.001	0.001	0.001
		Grilled	average	0.001	0.0005	0.0003	0.0002	0.0001	0.0001
			97.5th percentile	0.005	0.004	0.001	0.001	0.001	0.001
	Ornate rock lobster	Boiled	average	0.001	0.0005	0.0003	0.0002	0.0001	0.0001
			97.5th percentile	0.005	0.004	0.001	0.001	0.001	0.001
		Fried	average	0.014	0.009	0.006	0.004	0.002	0.002
			97.5th percentile	0.094	0.073	0.030	0.026	0.019	0.015
		Grilled	average	0.016	0.011	0.007	0.005	0.003	0.002
			97.5th percentile	0.113	0.087	0.036	0.031	0.023	0.017
Crabs (captured)	Musk Crab	Boiled	average	0.007	0.004	0.003	0.003	0.002	0.001
			97.5th percentile	0.060	0.044	0.019	0.025	0.016	0.009
		Fried	average	0.007	0.004	0.003	0.003	0.002	0.001
			97.5th percentile	0.060	0.044	0.019	0.025	0.016	0.009
		Grilled	average	0.008	0.005	0.003	0.004	0.003	0.001
			97.5th percentile	0.072	0.052	0.023	0.030	0.020	0.010
	Blue crab	Boiled	average	0.001	0.001	0.0003	0.0004	0.0003	0.0001
			97.5th percentile	0.008	0.006	0.003	0.003	0.002	0.001
		Fried	average	0.001	0.001	0.0003	0.000	0.0003	0.0001
			97.5th percentile	0.008	0.006	0.003	0.003	0.002	0.001
		Grilled	average	0.001	0.001	0.0005	0.001	0.0004	0.0001
			97.5th percentile	0.011	0.008	0.004	0.005	0.003	0.002

Table S4. Hazard quotient of mercury from each seafood species (Per capita). cont.

Type of sample		Cooking method	Food consumption	Age group (Year old)					
				3 to 5.9	6 to 12.9	13 to 17.9	18 to 34.9	35 to 64.9	65 and older
Crabs (captured)	Serrated Mud Crab	Boiled	average	0.002	0.001	0.001	0.001	0.001	0.0003
			97.5th percentile	0.021	0.015	0.007	0.009	0.006	0.003
		Fried	average	0.002	0.001	0.001	0.001	0.001	0.000
			97.5th percentile	0.021	0.015	0.007	0.009	0.006	0.003
		Grilled	average	0.002	0.001	0.001	0.001	0.001	0.000
			97.5th percentile	0.021	0.015	0.007	0.009	0.006	0.003
	Red frog crab	Boiled	average	0.001	0.001	0.0003	0.0004	0.0003	0.0001
			97.5th percentile	0.008	0.006	0.003	0.003	0.002	0.001
		Fried	average	0.001	0.001	0.0003	0.0004	0.0003	0.0001
			97.5th percentile	0.008	0.006	0.003	0.003	0.002	0.001
		Grilled	average	0.001	0.001	0.0003	0.0004	0.0003	0.0001
			97.5th percentile	0.008	0.006	0.003	0.003	0.002	0.001
Squids (captured)	Splendid squid	Boiled	average	0.009	0.008	0.006	0.005	0.002	0.001
			97.5th percentile	0.067	0.046	0.043	0.030	0.012	0.007
		Fried	average	0.009	0.008	0.006	0.005	0.002	0.001
			97.5th percentile	0.067	0.046	0.043	0.030	0.012	0.007
		Grilled	average	0.008	0.007	0.005	0.004	0.002	0.001
			97.5th percentile	0.058	0.040	0.038	0.027	0.011	0.006
	Cuttlefish	Boiled	average	0.026	0.022	0.016	0.013	0.006	0.003
			97.5th percentile	0.187	0.129	0.121	0.085	0.034	0.019
		Fried	average	0.026	0.022	0.016	0.013	0.006	0.003
			97.5th percentile	0.187	0.129	0.121	0.085	0.034	0.019
		Grilled	average	0.026	0.022	0.016	0.013	0.006	0.003
			97.5th percentile	0.187	0.129	0.121	0.085	0.034	0.019
	Bigfin reef squid	Boiled	average	0.010	0.009	0.006	0.005	0.002	0.001
			97.5th percentile	0.08	0.05	0.05	0.03	0.01	0.01
		Fried	average	0.010	0.009	0.006	0.005	0.002	0.001
			97.5th percentile	0.075	0.052	0.048	0.034	0.014	0.008
		Grilled	average	0.012	0.010	0.007	0.006	0.003	0.001
			97.5th percentile	0.088	0.060	0.057	0.040	0.016	0.009
Shellfish (captured)	Razor clam	Boiled	average	0.0001	0.0002	0.0001	0.0001	0.00005	0.00004
			97.5th percentile	-	0.00070	-	-	-	-
		Fried	average	0.0001	0.0002	0.0001	0.0001	0.00005	0.00004
			97.5th percentile	-	0.001	-	-	-	-
		Grilled	average	0.0001	0.0003	0.0001	0.0001	0.0001	0.00004
			97.5th percentile	-	0.001	-	-	-	-
	Oysters	Boiled	average	0.00002	0.00003	0.0001	0.0001	0.00004	0.00003
			97.5th percentile	-	-	0.001	0.001	0.0005	-
		Fried	average	0.00002	0.00003	0.0001	0.0001	0.00004	0.00003
			97.5th percentile	-	-	0.001	0.001	0.0005	-
		Grilled	average	0.0000	0.0001	0.0002	0.0002	0.0001	0.00004
			97.5th percentile	-	-	0.002	0.002	0.001	-

Table S4. Hazard quotient of mercury from each seafood species (Per capita). cont.

Type of sample		Cooking method	Food consumption	Age group (Year old)					
				3 to 5.9	6 to 12.9	13 to 17.9	18 to 34.9	35 to 64.9	65 and older
Shellfish (captured)	Cockle	Boiled	average	0.008	0.008	0.005	0.005	0.002	0.001
			97.5th percentile	0.044	0.091	0.057	0.048	0.024	0.007
		Fried	average	0.008	0.008	0.005	0.005	0.002	0.001
			97.5th percentile	0.044	0.091	0.057	0.048	0.024	0.007
		Grilled	average	0.021	0.019	0.013	0.012	0.006	0.002
			97.5th percentile	0.110	0.227	0.142	0.120	0.060	0.017
	Clam	Boiled	average	0.00004	0.00004	0.00003	0.00003	0.00003	0.00001
			97.5th percentile	0.0005	0.001	0.0003	0.0004	0.0003	0.0001
		Fried	average	0.00004	0.00004	0.00003	0.00003	0.00003	0.00001
			97.5th percentile	0.0005	0.001	0.0003	0.0004	0.0003	0.0001
		Grilled	average	0.0001	0.0001	0.00003	0.00004	0.00003	0.00002
			97.5th percentile	0.001	0.001	0.0004	0.0005	0.0003	0.0002
	Mussels	Boiled	average	0.001	0.001	0.0004	0.001	0.0003	0.0001
			97.5th percentile	0.011	0.006	0.003	0.004	0.003	0.001
		Fried	average	0.001	0.001	0.0004	0.001	0.0003	0.0001
			97.5th percentile	0.011	0.006	0.003	0.004	0.003	0.001
		Grilled	average	0.001	0.001	0.001	0.001	0.000	0.000
			97.5th percentile	0.018	0.009	0.006	0.007	0.005	0.002
	Wedge shell	Boiled	average	0.0001	0.0002	0.0001	0.0001	0.00005	0.00003
			97.5th percentile	-	0.001	-	-	-	-
		Fried	average	0.0001	0.0002	0.0001	0.0001	0.00005	0.00003
			97.5th percentile	-	0.001	-	-	-	-
		Grilled	average	0.0001	0.0002	0.0001	0.0001	0.00005	0.00003
			97.5th percentile	-	0.001	-	-	-	-
	Indo-Pacific horseshoe crab (eggs)	Boiled	average	0.0002	0.0003	0.0001	0.0000	0.0001	0.0001
			97.5th percentile	0.002	0.004	0.001	0.0002	0.001	0.001
		Fried	average	0.0002	0.0003	0.0001	0.00002	0.0001	0.0001
			97.5th percentile	0.002	0.004	0.001	0.0002	0.001	0.001
		Grilled	average	0.0002	0.0004	0.0001	0.00002	0.0001	0.0001
			97.5th percentile	0.002	0.005	0.001	0.0002	0.001	0.001
Marine fish (farmed)	Northern whiting fish	Boiled	average	0.002	0.001	0.001	0.001	0.001	0.001
			97.5th percentile	0.020	0.011	0.010	0.019	0.011	0.006
		Fried	average	0.002	0.001	0.001	0.001	0.001	0.001
			97.5th percentile	0.020	0.011	0.010	0.019	0.011	0.006
		Grilled	average	0.003	0.002	0.001	0.002	0.002	0.001
			97.5th percentile	0.031	0.016	0.014	0.028	0.017	0.009
	Silver pomfret	Boiled	average	0.0001	0.00005	0.00003	0.0001	0.00004	0.00003
			97.5th percentile	0.001	0.0004	0.0004	0.001	0.0004	0.0002
		Fried	average	0.0001	0.00005	0.00003	0.0001	0.00004	0.00003
			97.5th percentile	0.001	0.0004	0.0004	0.001	0.0004	0.0002
		Grilled	average	0.0001	0.0001	0.00003	0.0001	0.0001	0.00004
			97.5th percentile	0.001	0.001	0.0005	0.001	0.001	0.0003

Table S5. Hazard quotient of mercury from each seafood species (Per eater-only).

Type of sample		Cooking method	Food consumption	Age group (Year old)					
				3 to 5.9	6 to 12.9	13 to 17.9	18 to 34.9	35 to 64.9	65 and older
Shrimp and prawn (captured)	Pacific white shrimp	Boiled	average	0.008	0.005	0.003	0.003	0.002	0.002
			97.5th percentile	0.016	0.008	0.005	0.004	0.004	0.005
		Fried	average	0.006	0.004	0.003	0.002	0.002	0.002
			97.5th percentile	0.014	0.007	0.004	0.004	0.004	0.004
		Grilled	average	0.006	0.004	0.003	0.002	0.002	0.002
			97.5th percentile	0.014	0.007	0.004	0.004	0.004	0.004
	Banana prawn	Boiled	average	0.008	0.005	0.003	0.003	0.002	0.002
			97.5th percentile	0.016	0.008	0.005	0.004	0.004	0.005
		Fried	average	0.006	0.004	0.003	0.002	0.002	0.002
			97.5th percentile	0.014	0.007	0.004	0.004	0.004	0.004
		Grilled	average	0.006	0.004	0.003	0.002	0.002	0.002
			97.5th percentile	0.014	0.007	0.004	0.004	0.004	0.004
	Giant Tiger Prawn	Boiled	average	0.006	0.004	0.003	0.002	0.002	0.002
			97.5th percentile	0.014	0.007	0.004	0.004	0.004	0.004
		Fried	average	0.008	0.005	0.003	0.003	0.002	0.002
			97.5th percentile	0.016	0.008	0.005	0.004	0.004	0.005
		Grilled	average	0.008	0.005	0.003	0.003	0.002	0.002
			97.5th percentile	0.016	0.008	0.005	0.004	0.004	0.005
	Ornate rock lobster	Boiled	average	0.008	0.005	0.003	0.003	0.002	0.002
			97.5th percentile	0.016	0.008	0.005	0.004	0.004	0.005
		Fried	average	0.115	0.080	0.046	0.041	0.034	0.032
			97.5th percentile	0.246	0.127	0.080	0.067	0.067	0.076
		Grilled	average	0.183	0.127	0.073	0.066	0.055	0.051
			97.5th percentile	0.394	0.204	0.127	0.108	0.107	0.122
Crabs (captured)	Musk Crab	Boiled	average	0.289	0.185	0.130	0.133	0.137	0.108
			97.5th percentile	0.422	0.436	0.409	0.461	0.401	0.261
		Fried	average	0.433	0.277	0.195	0.199	0.206	0.163
			97.5th percentile	0.633	0.654	0.613	0.692	0.601	0.391
		Grilled	average	0.347	0.222	0.156	0.159	0.164	0.130
			97.5th percentile	0.506	0.523	0.490	0.553	0.481	0.313
	Blue crab	Boiled	average	0.038	0.024	0.017	0.017	0.018	0.014
			97.5th percentile	0.056	0.057	0.054	0.061	0.053	0.034
		Fried	average	0.053	0.034	0.024	0.024	0.025	0.020
			97.5th percentile	0.078	0.080	0.075	0.085	0.074	0.048
		Grilled	average	0.053	0.034	0.024	0.024	0.025	0.020
			97.5th percentile	0.078	0.080	0.075	0.085	0.074	0.048

Table S5. Hazard quotient of mercury from each seafood species (Per eater-only). cont.

Type of sample		Cooking method	Food consumption	Age group (Year old)					
				3 to 5.9	6 to 12.9	13 to 17.9	18 to 34.9	35 to 64.9	65 and older
Crabs (captured)	Serrated Mud Crab	Boiled	average	0.099	0.064	0.045	0.046	0.047	0.037
			97.5th percentile	0.145	0.150	0.141	0.159	0.138	0.090
		Fried	average	0.099	0.064	0.045	0.046	0.047	0.037
			97.5th percentile	0.145	0.150	0.141	0.159	0.138	0.090
		Grilled	average	0.099	0.064	0.045	0.046	0.047	0.037
			97.5th percentile	0.145	0.150	0.141	0.159	0.138	0.090
	Red frog crab	Boiled	average	0.037	0.024	0.017	0.017	0.018	0.014
			97.5th percentile	0.055	0.056	0.053	0.060	0.052	0.034
		Fried	average	0.044	0.028	0.020	0.020	0.021	0.016
			97.5th percentile	0.064	0.066	0.062	0.070	0.060	0.039
		Grilled	average	0.037	0.024	0.017	0.017	0.018	0.014
			97.5th percentile	0.055	0.056	0.053	0.060	0.052	0.034
Squids (captured)	Splendid squid	Boiled	average	0.102	0.072	0.045	0.042	0.035	0.029
			97.5th percentile	0.259	0.160	0.100	0.085	0.084	0.080
		Fried	average	0.142	0.101	0.063	0.059	0.049	0.041
			97.5th percentile	0.362	0.225	0.140	0.119	0.118	0.112
		Grilled	average	0.089	0.063	0.039	0.037	0.031	0.026
			97.5th percentile	0.226	0.140	0.088	0.074	0.074	0.070
	Cuttlefish	Boiled	average	0.287	0.203	0.127	0.118	0.098	0.082
			97.5th percentile	0.729	0.452	0.282	0.239	0.237	0.225
		Fried	average	0.401	0.284	0.177	0.165	0.138	0.115
			97.5th percentile	1.020	0.633	0.395	0.335	0.332	0.315
		Grilled	average	0.287	0.203	0.127	0.118	0.098	0.082
			97.5th percentile	0.729	0.452	0.282	0.239	0.237	0.225
	Bigfin reef squid	Boiled	average	0.115	0.081	0.051	0.047	0.039	0.033
			97.5th percentile	0.292	0.181	0.113	0.096	0.095	0.090
		Fried	average	0.134	0.095	0.059	0.055	0.046	0.039
			97.5th percentile	0.341	0.211	0.132	0.112	0.111	0.105
		Grilled	average	0.134	0.095	0.059	0.055	0.046	0.039
			97.5th percentile	0.341	0.211	0.132	0.112	0.111	0.105
Shellfish (captured)	Razor clam	Boiled	average	0.080	0.040	0.026	0.032	0.023	0.019
			97.5th percentile	0.162	0.084	0.052	0.067	0.044	0.025
		Fried	average	0.092	0.046	0.030	0.036	0.026	0.021
			97.5th percentile	0.186	0.096	0.060	0.076	0.050	0.029
		Grilled	average	0.092	0.046	0.030	0.036	0.026	0.021
			97.5th percentile	0.186	0.096	0.060	0.076	0.050	0.029
	Oysters	Boiled	average	0.027	0.023	0.017	0.014	0.012	0.011
			97.5th percentile	0.103	0.027	0.033	0.028	0.028	0.016
		Fried	average	0.033	0.029	0.021	0.018	0.015	0.013
			97.5th percentile	0.129	0.033	0.042	0.035	0.035	0.020
		Grilled	average	0.044	0.038	0.028	0.024	0.020	0.018
			97.5th percentile	0.172	0.044	0.055	0.047	0.047	0.027

Table S5. Hazard quotient of mercury from each seafood species (Per eater-only). cont.

Type of sample		Cooking method	Food consumption	Age group (Year old)					
				3 to 5.9	6 to 12.9	13 to 17.9	18 to 34.9	35 to 64.9	65 and older
Shellfish (captured)	Cockle	Boiled	average	0.287	0.195	0.128	0.116	0.110	0.094
			97.5th percentile	0.616	0.637	0.199	0.337	0.251	0.191
		Fried	average	0.359	0.244	0.159	0.146	0.137	0.117
			97.5th percentile	0.770	0.796	0.249	0.421	0.314	0.238
		Grilled	average	0.718	0.488	0.319	0.291	0.274	0.234
			97.5th percentile	**1.541**	**1.593**	0.498	0.842	0.628	0.477
	Clam	Boiled	average	0.006	0.004	0.003	0.002	0.002	0.002
			97.5th percentile	0.014	0.007	0.007	0.004	0.004	0.003
		Fried	average	0.008	0.005	0.004	0.004	0.003	0.003
			97.5th percentile	0.021	0.011	0.010	0.006	0.006	0.005
		Grilled	average	0.007	0.004	0.003	0.003	0.002	0.002
			97.5th percentile	0.017	0.009	0.008	0.005	0.005	0.004
	Mussels	Boiled	average	0.032	0.022	0.015	0.014	0.013	0.012
			97.5th percentile	0.075	0.039	0.024	0.041	0.051	0.023
		Fried	average	0.053	0.037	0.025	0.023	0.022	0.019
			97.5th percentile	0.125	0.065	0.040	0.068	0.085	0.039
		Grilled	average	0.053	0.037	0.025	0.023	0.022	0.019
			97.5th percentile	0.125	0.065	0.040	0.068	0.085	0.039
	Wedge shell	Boiled	average	0.078	0.039	0.026	0.031	0.022	0.018
			97.5th percentile	0.158	0.081	0.051	0.065	0.043	0.024
		Fried	average	0.094	0.047	0.031	0.037	0.027	0.022
			97.5th percentile	0.189	0.098	0.061	0.078	0.051	0.029
		Grilled	average	0.078	0.039	0.026	0.031	0.022	0.018
			97.5th percentile	0.158	0.081	0.051	0.065	0.043	0.024
	Indo-Pacific horseshoe crab (eggs)	Boiled	average	0.028	0.019	0.012	0.002	0.009	0.010
			97.5th percentile	0.057	0.059	0.037	0.004	0.015	0.035
		Fried	average	0.036	0.025	0.016	0.002	0.012	0.013
			97.5th percentile	0.073	0.076	0.047	0.006	0.020	0.045
		Grilled	average	0.031	0.022	0.014	0.002	0.010	0.011
			97.5th percentile	0.064	0.066	0.041	0.005	0.017	0.040
Marine fish (farmed)	Northern whiting fish	Boiled	average	0.085	0.062	0.039	0.037	0.032	0.030
			97.5th percentile	0.143	0.148	0.092	0.078	0.078	0.085
		Fried	average	0.152	0.112	0.071	0.066	0.058	0.054
			97.5th percentile	0.257	0.266	0.166	0.141	0.140	0.153
		Grilled	average	0.127	0.094	0.059	0.055	0.048	0.045
			97.5th percentile	0.215	0.222	0.139	0.117	0.117	0.127
	Silver pomfret	Boiled	average	0.003	0.002	0.002	0.001	0.001	0.001
			97.5th percentile	0.006	0.006	0.004	0.003	0.003	0.003
		Fried	average	0.006	0.004	0.003	0.003	0.002	0.002
			97.5th percentile	0.010	0.010	0.007	0.006	0.006	0.006
		Grilled	average	0.004	0.003	0.002	0.002	0.002	0.002
			97.5th percentile	0.007	0.007	0.005	0.004	0.004	0.004

Table S6. Margin of exposure of lead from each seafood species (Per capita).

Type of sample		Cooking method	Food consumption	Age group (Year old)					
				3 to 5.9	6 to 12.9	13 to 17.9	18 to 34.9	35 to 64.9	65 and older
Shrimp and prawn (captured)	Pacific white shrimp	Boiled	average	**65.67**	**96.53**	156.29	262.85	454.24	704.46
			97.5th percentile	**9.46**	**12.20**	**29.29**	**43.60**	**58.52**	**77.07**
		Fried	average	**76.62**	112.62	182.33	306.66	529.94	821.87
			97.5th percentile	**11.03**	**14.23**	**34.17**	**50.87**	**68.27**	**89.92**
		Grilled	average	**76.62**	112.62	182.33	306.66	529.94	821.87
			97.5th percentile	**11.03**	**14.23**	**34.17**	**50.87**	**68.27**	**89.92**
	Banana prawn	Boiled	average	**74.69**	109.79	177.76	298.96	516.65	801.26
			97.5th percentile	**10.76**	**13.87**	**33.31**	**49.59**	**66.56**	**87.66**
		Fried	average	**87.14**	128.09	207.39	348.79	602.76	934.80
			97.5th percentile	**12.55**	**16.19**	**38.86**	**57.86**	**77.65**	102.28
		Grilled	average	**87.14**	128.09	207.39	348.79	602.76	934.80
			97.5th percentile	**12.55**	**16.19**	**38.86**	**57.86**	**77.65**	102.28
	Giant Tiger Prawn	Boiled	average	106.59	156.68	253.66	426.62	737.26	1143.40
			97.5th percentile	**15.35**	**19.80**	**47.53**	**70.77**	**94.98**	125.10
		Fried	average	**91.36**	134.29	217.43	365.68	631.94	980.06
			97.5th percentile	**13.16**	**16.97**	**40.74**	**60.66**	**81.41**	107.23
		Grilled	average	**91.36**	134.29	217.43	365.68	631.94	980.06
			97.5th percentile	**13.16**	**16.97**	**40.74**	**60.66**	**81.41**	107.23
	Ornate rock lobster	Boiled	average	120.20	176.68	286.05	481.08	831.38	1289.36
			97.5th percentile	**17.31**	**22.33**	**53.60**	**79.80**	107.11	141.07
		Fried	average	160.26	235.57	381.39	641.45	1108.50	1719.15
			97.5th percentile	**23.08**	**29.77**	**71.47**	106.40	142.81	188.09
		Grilled	average	100.16	147.23	238.37	400.90	692.81	1074.47
			97.5th percentile	**14.42**	**18.61**	**44.67**	**66.50**	**89.25**	117.56
Crabs (captured)	Musk Crab	Boiled	average	153.19	253.05	409.97	415.46	578.61	1617.86
			97.5th percentile	**17.05**	**23.58**	**52.79**	**52.43**	**79.11**	149.09
		Fried	average	102.13	168.70	273.31	276.97	385.74	1078.57
			97.5th percentile	**11.37**	**15.72**	**35.20**	**34.95**	**52.74**	**99.39**
		Grilled	average	127.66	210.88	341.64	346.21	482.17	1348.22
			97.5th percentile	**14.21**	**19.65**	**44.00**	**43.69**	**65.93**	124.24
	Blue crab	Boiled	average	232.61	384.24	622.51	630.84	878.58	2456.61
			97.5th percentile	**25.89**	**35.80**	**80.16**	**79.61**	120.12	226.38
		Fried	average	166.15	274.46	444.65	450.60	627.55	1754.72
			97.5th percentile	**18.49**	**25.57**	**57.26**	**56.86**	**85.80**	161.70
		Grilled	average	166.15	274.46	444.65	450.60	627.55	1754.72
			97.5th percentile	**18.49**	**25.57**	**57.26**	**56.86**	**85.80**	161.70

Table S6. Margin of exposure of lead from each seafood species (Per capita). cont.

Type of sample		Cooking method	Food consumption	Age group (Year old)					
				3 to 5.9	6 to 12.9	13 to 17.9	18 to 34.9	35 to 64.9	65 and older
Crabs (captured)	Serrated Mud Crab	Boiled	average	171.06	282.58	457.81	463.93	646.12	1806.64
			97.5th percentile	**19.04**	**26.33**	**58.95**	**58.55**	**88.34**	166.48
		Fried	average	171.06	282.58	457.81	463.93	646.12	1806.64
			97.5th percentile	**19.04**	**26.33**	**58.95**	**58.55**	**88.34**	166.48
		Grilled	average	171.06	282.58	457.81	463.93	646.12	1806.64
			97.5th percentile	**19.04**	**26.33**	**58.95**	**58.55**	**88.34**	166.48
	Red frog crab	Boiled	average	**41.80**	**69.05**	111.87	113.37	157.89	441.48
			97.5th percentile	**4.65**	**6.43**	**14.41**	**14.31**	**21.59**	**40.68**
		Fried	average	**35.83**	**59.19**	**95.89**	**97.17**	135.33	378.41
			97.5th percentile	**3.99**	**5.52**	**12.35**	**12.26**	**18.50**	**34.87**
		Grilled	average	**41.80**	**69.05**	111.87	113.37	157.89	441.48
			97.5th percentile	**4.65**	**6.43**	**14.41**	**14.31**	**21.59**	**40.68**
Squids (captured)	Splendid squid	Boiled	average	103.77	121.96	169.45	247.15	571.29	1329.85
			97.5th percentile	**14.12**	**20.50**	**21.87**	**39.07**	**98.27**	172.53
		Fried	average	**74.12**	**87.11**	121.03	176.54	408.07	949.90
			97.5th percentile	**10.09**	**14.64**	**15.62**	**27.90**	**70.19**	123.24
		Grilled	average	118.59	139.38	193.65	282.46	652.91	1519.83
			97.5th percentile	**16.14**	**23.42**	**25.00**	**44.65**	112.31	197.18
	Cuttlefish	Boiled	average	**45.39**	**53.34**	**74.11**	108.10	249.87	581.65
			97.5th percentile	**6.18**	**8.96**	**9.57**	**17.09**	**42.98**	**75.46**
		Fried	average	**32.42**	**38.10**	**52.94**	**77.21**	178.48	415.47
			97.5th percentile	**4.41**	**6.40**	**6.83**	**12.20**	**30.70**	**53.90**
		Grilled	average	**45.39**	**53.34**	**74.11**	108.10	249.87	581.65
			97.5th percentile	**6.18**	**8.96**	**9.57**	**17.09**	**42.98**	**75.46**
	Bigfin reef squid	Boiled	average	183.51	215.68	299.67	437.09	1010.34	2351.86
			97.5th percentile	**24.98**	**36.25**	**38.68**	**69.09**	173.79	305.12
		Fried	average	157.29	184.87	256.86	374.65	866.01	2015.88
			97.5th percentile	**21.41**	**31.07**	**33.15**	**59.22**	148.96	261.54
		Grilled	average	157.29	184.87	256.86	374.65	866.01	2015.88
			97.5th percentile	**21.41**	**31.07**	**33.15**	**59.22**	148.96	261.54
Shellfish (captured)	Razor clam	Boiled	average	1096.28	530.35	2263.32	2888.24	3391.50	4465.85
			97.5th percentile	-	180.54	-	-	-	-
		Fried	average	959.25	464.05	1980.40	2527.21	2967.56	3907.62
			97.5th percentile	-	157.98	-	-	-	-
		Grilled	average	959.25	464.05	1980.40	2527.21	2967.56	3907.62
			97.5th percentile	-	157.98	-	-	-	-
	Oysters	Boiled	average	972.47	470.45	115.83	142.34	451.27	720.27
			97.5th percentile	-	-	**13.69**	**13.59**	**41.02**	-
		Fried	average	777.97	376.36	**92.66**	113.87	361.01	576.21
			97.5th percentile	-	-	**10.95**	**10.87**	**32.82**	-
		Grilled	average	583.48	282.27	**69.50**	**85.40**	270.76	432.16
			97.5th percentile	-	-	**8.21**	**8.15**	**24.61**	-

Table S6. Margin of exposure of lead from each seafood species (Per capita). cont.

Type of sample		Cooking method	Food consumption	Age group (Year old)					
				3 to 5.9	6 to 12.9	13 to 17.9	18 to 34.9	35 to 64.9	65 and older
Shellfish (captured)	Cockle	Boiled	average	**28.34**	**31.11**	**46.77**	**61.52**	125.05	349.92
			97.5th percentile	**5.35**	**2.59**	**4.14**	**6.16**	**12.40**	**43.57**
		Fried	average	**22.67**	**24.88**	**37.42**	**49.22**	100.04	279.93
			97.5th percentile	**4.28**	**2.07**	**3.31**	**4.93**	**9.92**	**34.86**
		Grilled	average	**11.34**	**12.44**	**18.71**	**24.61**	**50.02**	139.97
			97.5th percentile	**2.14**	**1.03**	**1.66**	**2.46**	**4.96**	**17.43**
	Clam	Boiled	average	1411.62	1392.57	2185.76	2256.20	2747.01	6229.63
			97.5th percentile	125.26	121.20	193.96	206.36	290.64	547.66
		Fried	average	941.08	928.38	1457.17	1504.14	1831.34	4153.09
			97.5th percentile	**83.51**	**80.80**	129.31	137.57	193.76	365.11
		Grilled	average	1176.35	1160.48	1821.47	1880.17	2289.17	5191.36
			97.5th percentile	104.39	101.00	161.63	171.97	242.20	456.38
	Mussels	Boiled	average	**64.39**	**79.90**	141.74	119.96	223.15	573.16
			97.5th percentile	**4.75**	**9.19**	**14.71**	**14.60**	**22.04**	**55.27**
		Fried	average	**38.63**	**47.94**	**85.04**	**71.98**	133.89	343.90
			97.5th percentile	**2.85**	**5.51**	**8.82**	**8.76**	**13.22**	**33.16**
		Grilled	average	**38.63**	**47.94**	**85.04**	**71.98**	133.89	343.90
			97.5th percentile	**2.85**	**5.51**	**8.82**	**8.76**	**13.22**	**33.16**
	Wedge shell	Boiled	average	1983.97	959.78	4096.00	5226.93	6137.69	8081.99
			97.5th percentile	-	326.73	-	-	-	-
		Fried	average	1653.31	799.82	3413.33	4355.77	5114.74	6734.99
			97.5th percentile	-	272.28	-	-	-	-
		Grilled	average	1983.97	959.78	4096.00	5226.93	6137.69	8081.99
			97.5th percentile	-	326.73	-	-	-	-
	Indo-Pacific horseshoe crab (eggs)	Boiled	average	1013.99	490.54	1449.29	1753.13	2352.69	2253.07
			97.5th percentile	**83.34**	**37.49**	128.17	178.66	192.06	168.60
		Fried	average	788.66	381.53	1127.23	1363.54	1829.87	1752.39
			97.5th percentile	**64.82**	**29.16**	**99.69**	138.96	149.38	131.13
		Grilled	average	901.32	436.03	1288.26	1558.34	2091.28	2002.73
			97.5th percentile	**74.08**	**33.33**	113.93	158.81	170.72	149.86
Marine fish (farmed)	Northern whiting fish	Boiled	average	162.38	274.45	488.71	303.87	358.58	518.67
			97.5th percentile	**15.69**	**30.37**	**33.72**	**21.52**	**36.38**	**63.93**
		Fried	average	**90.21**	152.47	271.50	168.82	199.21	288.15
			97.5th percentile	**8.72**	**16.87**	**18.73**	**11.96**	**20.21**	**35.51**
		Grilled	average	108.26	182.97	325.81	202.58	239.05	345.78
			97.5th percentile	**10.46**	**20.24**	**22.48**	**14.35**	**24.26**	**42.62**
	Silver pomfret	Boiled	average	1225.49	2071.23	3688.21	2293.29	2706.15	3914.34
			97.5th percentile	118.43	229.17	254.48	162.44	274.59	482.44
		Fried	average	680.83	1150.69	2049.00	1274.05	1503.41	2174.63
			97.5th percentile	**65.79**	127.32	141.38	**90.25**	152.55	268.02
		Grilled	average	953.16	1610.96	2868.61	1783.67	2104.78	3044.49
			97.5th percentile	**92.11**	178.24	197.93	126.34	213.57	375.23

Table S7. Margin of exposure of lead from each seafood species (Per eater-only).

Type of sample		Cooking method	Food consumption	Age group (Year old)					
				3 to 5.9	6 to 12.9	13 to 17.9	18 to 34.9	35 to 64.9	65 and older
Shrimp and prawn (captured)	Pacific white shrimp	Boiled	average	**5.81**	**8.37**	**14.56**	**20.23**	**24.55**	**26.41**
			97.5th percentile	**2.70**	**5.23**	**8.37**	**12.46**	**12.54**	**11.01**
		Fried	average	**6.78**	**9.76**	**16.99**	**23.60**	**28.64**	**30.81**
			97.5th percentile	**3.15**	**6.10**	**9.76**	**14.53**	**14.63**	**12.84**
		Grilled	average	**6.78**	**9.76**	**16.99**	**23.60**	**28.64**	**30.81**
			97.5th percentile	**3.15**	**6.10**	**9.76**	**14.53**	**14.63**	**12.84**
	Banana prawn	Boiled	average	**6.61**	**9.52**	**16.56**	**23.00**	**27.92**	**30.04**
			97.5th percentile	**3.07**	**5.95**	**9.52**	**14.17**	**14.26**	**12.52**
		Fried	average	**7.71**	**11.10**	**19.32**	**26.84**	**32.57**	**35.05**
			97.5th percentile	**3.59**	**6.94**	**11.10**	**16.53**	**16.64**	**14.61**
		Grilled	average	**7.71**	**11.10**	**19.32**	**26.84**	**32.57**	**35.05**
			97.5th percentile	**3.59**	**6.94**	**11.10**	**16.53**	**16.64**	**14.61**
	Giant Tiger Prawn	Boiled	average	**9.43**	**13.58**	**23.64**	**32.83**	**39.84**	**42.87**
			97.5th percentile	**4.39**	**8.49**	**13.58**	**20.22**	**20.35**	**17.87**
		Fried	average	**8.09**	**11.64**	**20.26**	**28.14**	**34.15**	**36.74**
			97.5th percentile	**3.76**	**7.27**	**11.64**	**17.33**	**17.44**	**15.31**
		Grilled	average	**2.70**	**5.23**	**8.37**	**12.46**	**12.54**	**11.01**
			97.5th percentile	**3.76**	**7.27**	**11.64**	**17.33**	**17.44**	**15.31**
	Ornate rock lobster	Boiled	average	**10.64**	**15.32**	**26.65**	**37.02**	**44.93**	**48.34**
			97.5th percentile	**4.95**	**9.57**	**15.32**	**22.80**	**22.95**	**20.15**
		Fried	average	**14.18**	**20.42**	**35.54**	**49.36**	**59.90**	**64.45**
			97.5th percentile	**6.59**	**12.76**	**20.42**	**30.40**	**30.60**	**26.86**
		Grilled	average	**8.86**	**12.76**	**22.21**	**30.85**	**37.44**	**40.28**
			97.5th percentile	**4.12**	**7.97**	**12.76**	**19.00**	**19.12**	**16.79**
Crabs (captured)	Musk Crab	Boiled	average	**3.56**	**5.57**	**7.93**	**9.77**	**9.45**	**11.95**
			97.5th percentile	**2.44**	**2.36**	**2.52**	**2.81**	**3.23**	**4.96**
		Fried	average	**2.37**	**3.71**	**5.28**	**6.52**	**6.30**	**7.97**
			97.5th percentile	**1.62**	**1.57**	**1.68**	**1.87**	**2.15**	**3.31**
		Grilled	average	**2.97**	**4.64**	**6.60**	**8.14**	**7.87**	**9.96**
			97.5th percentile	**2.03**	**1.96**	**2.10**	**2.34**	**2.69**	**4.14**
	Blue crab	Boiled	average	**5.40**	**8.45**	**12.03**	**14.84**	**14.35**	**18.15**
			97.5th percentile	**3.70**	**3.58**	**3.82**	**4.27**	**4.91**	**7.54**
		Fried	average	**3.86**	**6.04**	**8.60**	**10.60**	**10.25**	**12.96**
			97.5th percentile	**2.64**	**2.56**	**2.73**	**3.05**	**3.50**	**5.38**
		Grilled	average	**3.86**	**6.04**	**8.60**	**10.60**	**10.25**	**12.96**
			97.5th percentile	**2.64**	**2.56**	**2.73**	**3.05**	**3.50**	**5.38**

Table S7. Margin of exposure of lead from each seafood species (Per eater-only). cont.

Type of sample		Cooking method	Food consumption	Age group (Year old)					
				3 to 5.9	6 to 12.9	13 to 17.9	18 to 34.9	35 to 64.9	65 and older
Crabs (captured)	Serrated Mud Crab	Boiled	average	**3.97**	**6.22**	**8.85**	**10.91**	**10.55**	**13.35**
			97.5th percentile	**2.72**	**2.63**	**2.81**	**3.14**	**3.61**	**5.54**
		Fried	average	**3.97**	**6.22**	**8.85**	**10.91**	**10.55**	**13.35**
			97.5th percentile	**2.72**	**2.63**	**2.81**	**3.14**	**3.61**	**5.54**
		Grilled	average	**3.97**	**6.22**	**8.85**	**10.91**	**10.55**	**13.35**
			97.5th percentile	**2.72**	**2.63**	**2.81**	**3.14**	**3.61**	**5.54**
	Red frog crab	Boiled	average	**0.97**	**1.52**	**2.16**	**2.67**	**2.58**	**3.26**
			97.5th percentile	**0.67**	**0.64**	**0.69**	**0.77**	**0.88**	**1.35**
		Fried	average	**0.83**	**1.30**	**1.85**	**2.29**	**2.21**	**2.80**
			97.5th percentile	**0.57**	**0.55**	**0.59**	**0.66**	**0.76**	**1.16**
		Grilled	average	**0.97**	**1.52**	**2.16**	**2.67**	**2.58**	**3.26**
			97.5th percentile	**0.67**	**0.64**	**0.69**	**0.77**	**0.88**	**1.35**
Squids (captured)	Splendid squid	Boiled	average	**9.23**	**13.03**	**20.90**	**28.27**	**33.87**	**40.44**
			97.5th percentile	**3.63**	**5.86**	**9.37**	**13.95**	**14.04**	**14.79**
		Fried	average	**6.59**	**9.31**	**14.93**	**20.19**	**24.19**	**28.88**
			97.5th percentile	**2.59**	**4.18**	**6.69**	**9.97**	**10.03**	**10.57**
		Grilled	average	**10.55**	**14.89**	**23.89**	**32.30**	**38.71**	**46.21**
			97.5th percentile	**4.15**	**6.69**	**10.71**	**15.95**	**16.05**	**16.91**
	Cuttlefish	Boiled	average	**4.04**	**5.70**	**9.14**	**12.36**	**14.81**	**17.69**
			97.5th percentile	**1.59**	**2.56**	**4.10**	**6.10**	**6.14**	**6.47**
		Fried	average	**2.88**	**4.07**	**6.53**	**8.83**	**10.58**	**12.63**
			97.5th percentile	**1.13**	**1.83**	**2.93**	**4.36**	**4.39**	**4.62**
		Grilled	average	**4.04**	**5.70**	**9.14**	**12.36**	**14.81**	**17.69**
			97.5th percentile	**1.59**	**2.56**	**4.10**	**6.10**	**6.14**	**6.47**
	Bigfin reef squid	Boiled	average	**16.33**	**23.05**	**36.96**	**49.99**	**59.89**	**71.51**
			97.5th percentile	**6.42**	**10.36**	**16.58**	**24.68**	**24.84**	**26.16**
		Fried	average	**14.00**	**19.76**	**31.68**	**42.85**	**51.34**	**61.30**
			97.5th percentile	**5.51**	**8.88**	**14.21**	**21.15**	**21.29**	**22.43**
		Grilled	average	**14.00**	**19.76**	**31.68**	**42.85**	**51.34**	**61.30**
			97.5th percentile	**5.51**	**8.88**	**14.21**	**21.15**	**21.29**	**22.43**
Shellfish (captured)	Razor clam	Boiled	average	**1.58**	**3.17**	**4.81**	**5.04**	**6.97**	**8.63**
			97.5th percentile	**0.78**	**1.52**	**2.42**	**2.41**	**3.63**	**6.38**
		Fried	average	**1.38**	**2.77**	**4.21**	**4.41**	**6.10**	**7.55**
			97.5th percentile	**0.69**	**1.33**	**2.12**	**2.11**	**3.18**	**5.58**
		Grilled	average	**1.38**	**2.77**	**4.21**	**4.41**	**6.10**	**7.55**
			97.5th percentile	**0.69**	**1.33**	**2.12**	**2.11**	**3.18**	**5.58**
	Oysters	Boiled	average	**0.57**	**0.66**	**0.92**	**1.35**	**1.60**	**1.80**
			97.5th percentile	**0.15**	**0.57**	**0.46**	**0.68**	**0.68**	**1.20**
		Fried	average	**0.46**	**0.53**	**0.74**	**1.08**	**1.28**	**1.44**
			97.5th percentile	**0.12**	**0.46**	**0.37**	**0.54**	**0.55**	**0.96**
		Grilled	average	**0.34**	**0.40**	**0.55**	**0.81**	**0.96**	**1.08**
			97.5th percentile	**0.09**	**0.34**	**0.27**	**0.41**	**0.41**	**0.72**

Table S7. Margin of exposure of lead from each seafood species (Per eater-only). cont.

Type of sample		Cooking method	Food consumption	Age group (Year old)					
				3 to 5.9	6 to 12.9	13 to 17.9	18 to 34.9	35 to 64.9	65 and older
Shellfish (captured)	Cockle	Boiled	average	**0.82**	**1.20**	**1.84**	**2.55**	**2.70**	**3.16**
			97.5th percentile	**0.38**	**0.37**	**1.18**	**0.88**	**1.18**	**1.56**
		Fried	average	**0.66**	**0.96**	**1.48**	**2.04**	**2.16**	**2.53**
			97.5th percentile	**0.31**	**0.30**	**0.95**	**0.70**	**0.94**	**1.24**
		Grilled	average	**0.33**	**0.48**	**0.74**	**1.02**	**1.08**	**1.26**
			97.5th percentile	**0.15**	**0.15**	**0.47**	**0.35**	**0.47**	**0.62**
	Clam	Boiled	average	**11.24**	**17.06**	**24.11**	**33.39**	**39.43**	**45.81**
			97.5th percentile	**4.48**	**8.66**	**9.24**	**20.64**	**20.77**	**24.31**
		Fried	average	**7.50**	**11.38**	**16.07**	**22.26**	**26.29**	**30.54**
			97.5th percentile	**2.98**	**5.77**	**6.16**	**13.76**	**13.85**	**16.21**
		Grilled	average	**9.37**	**14.22**	**20.09**	**27.82**	**32.86**	**38.17**
			97.5th percentile	**3.73**	**7.22**	**7.70**	**17.20**	**17.31**	**20.26**
	Mussels	Boiled	average	**1.61**	**2.30**	**3.41**	**4.57**	**4.86**	**5.54**
			97.5th percentile	**0.68**	**1.31**	**2.10**	**1.56**	**1.26**	**2.76**
		Fried	average	**0.97**	**1.38**	**2.05**	**2.74**	**2.92**	**3.33**
			97.5th percentile	**0.41**	**0.79**	**1.26**	**0.94**	**0.76**	**1.66**
		Grilled	average	**0.97**	**1.38**	**2.05**	**2.74**	**2.92**	**3.33**
			97.5th percentile	**0.41**	**0.79**	**1.26**	**0.94**	**0.76**	**1.66**
	Wedge shell	Boiled	average	**2.86**	**5.74**	**8.71**	**9.12**	**12.62**	**15.61**
			97.5th percentile	**1.42**	**2.74**	**4.39**	**4.36**	**6.58**	**11.55**
		Fried	average	**2.38**	**4.78**	**7.26**	**7.60**	**10.52**	**13.01**
			97.5th percentile	**1.18**	**2.29**	**3.66**	**3.63**	**5.48**	**9.62**
		Grilled	average	**2.86**	**5.74**	**8.71**	**9.12**	**12.62**	**15.61**
			97.5th percentile	**1.42**	**2.74**	**4.39**	**4.36**	**6.58**	**11.55**
	Indo-Pacific horseshoe crab (eggs)	Boiled	average	**5.70**	**8.19**	**12.92**	**18.64**	**21.97**	**19.73**
			97.5th percentile	**2.77**	**2.68**	**4.28**	**6.38**	**12.83**	**5.63**
		Fried	average	**4.43**	**6.37**	**10.05**	**14.50**	**17.09**	**15.35**
			97.5th percentile	**2.15**	**2.08**	**3.33**	**4.96**	**9.98**	**4.38**
		Grilled	average	**5.07**	**7.28**	**11.49**	**16.57**	**19.53**	**17.54**
			97.5th percentile	**2.46**	**2.38**	**3.81**	**5.67**	**11.41**	**5.01**
Marine fish (farmed)	Northern whiting fish	Boiled	average	**3.79**	**5.14**	**8.12**	**11.00**	**12.60**	**13.53**
			97.5th percentile	**2.24**	**2.17**	**3.47**	**5.17**	**5.20**	**4.75**
		Fried	average	**2.11**	**2.86**	**4.51**	**6.11**	**7.00**	**7.52**
			97.5th percentile	**1.24**	**1.20**	**1.93**	**2.87**	**2.89**	**2.64**
		Grilled	average	**2.53**	**3.43**	**5.41**	**7.34**	**8.40**	**9.02**
			97.5th percentile	**1.49**	**1.45**	**2.31**	**3.44**	**3.47**	**3.17**
	Silver pomfret	Boiled	average	**28.62**	**38.79**	**61.27**	**83.04**	**95.13**	102.09
			97.5th percentile	**16.91**	**16.36**	**26.19**	**38.99**	**39.24**	**35.88**
		Fried	average	**15.90**	**21.55**	**34.04**	**46.13**	**52.85**	**56.72**
			97.5th percentile	**9.40**	**9.09**	**14.55**	**21.66**	**21.80**	**19.93**
		Grilled	average	**22.26**	**30.17**	**47.65**	**64.58**	**73.99**	**79.41**
			97.5th percentile	**13.15**	**12.73**	**20.37**	**30.32**	**30.52**	**27.91**

Table S8. Cooking yield factor of each seafood species

Common name	cooking yield factor		
	**boiled**	**Fried**	**Grilled**
Pacific white shrimp	0.6	0.7	0.7
Banana prawn	0.6	0.7	0.7
Giant Tiger Prawn	0.7	0.6	0.6
Ornate rock lobster	0.6	0.8	0.5
Musk Crab	0.6	0.4	0.5
Blue crab	0.7	0.5	0.5
Serrated Mud Crab	0.7	0.7	0.7
Red frog crab	0.7	0.6	0.7
Splendid squid	0.7	0.5	0.8
Cuttlefish	0.7	0.5	0.7
Bigfin reef squid	0.7	0.6	0.6
Razor clam	0.8	0.7	0.7
Oysters	0.5	0.4	0.3
Cockle	0.5	0.4	0.2
Clam	0.6	0.4	0.5
Mussels	0.5	0.3	0.3
Wedge shell	0.6	0.5	0.6
Indo-Pacific horseshoe crab (eggs)	0.9	0.7	0.8
Northern whiting fish	0.9	0.5	0.6
Silver pomfret	0.9	0.5	0.7

Source: Rueangsri, N.; Judprasong, K.; Sridonpai, P.; Laitip, N.; Feldmann, J.; Singhato, A. Influence of Various Cooking Methods on Selenium Concentrations in Commonly Consumed Seafood Species in Thailand. *Foods* **2025**, *14*, 2700. https://doi.org/10.3390/foods14152700

The concentration of heavy metals in the cooked sample = (concentration of heavy metals in the raw sample / cooking yield factor)
